# Correlations between carotid plaque progression and mechanical stresses change sign over time: a patient follow up study using MRI and 3D FSI models

**DOI:** 10.1186/1475-925X-12-105

**Published:** 2013-10-14

**Authors:** Dalin Tang, Chun Yang, Gador Canton, Zheyang Wu, Thomas Hatsukami, Chun Yuan

**Affiliations:** 1 School of Biological Sciences and Medical Engineering, Southeast University, Nanjing, China; 2 Mathematical Sciences Department, Worcester Polytechnic Institute, 100 Institute Road, Worcester, MA 01609, USA; 3 China United Network Communications Co., Ltd., Beijing, China; 4 Department of Mechanical Engineering, University of Washington, Seattle, WA 98195, USA; 5 Division of Vascular Surgery, University of Washington, Seattle, WA 98195, USA; 6 Deparment of Radiology, University of Washington, Seattle, WA 98195, USA

**Keywords:** Plaque progression, Flow shear stress, Atherosclerosis, Carotid, Fluid–structure interaction

## Abstract

**Background:**

Increasing evidence suggests that mechanisms governing advanced plaque progression may be different from those for early progression and require further investigation. Serial MRI data and 3D fluid–structure interaction (FSI) models were employed to identify possible correlations between mechanical stresses and advanced plaque progression measured by vessel wall thickness increase (WTI). Long-term patient follow up was used to gather data and investigate if the correlations identified above were reproducible.

**Methods:**

In vivo MRI data were acquired from 16 patients in a follow-up study with 2 to 4 scans for each patient (scan interval: average 18 months and standard deviation 6.8 months). A total of 38 scan pairs (baseline and follow-up) were formed for analysis using the carotid bifurcation as the registration point. 3D FSI models were constructed to obtain plaque wall stress (PWS) and flow shear stress (FSS) to quantify their correlations with plaque progression. The Linear Mixed-Effects models were used to study possible correlations between WTI and baseline PWS and FSS with nodal dependence taken into consideration.

**Results:**

Of the 38 scan pairs, 22 pairs showed positive correlation between baseline PWS and WTI, 1 pair showed negative correlation, and 15 pairs showed no correlation. Thirteen patients changed their correlation sign (81.25%). Between baseline FSS and WTI, 16 pairs showed negative correlation, 1 pair showed positive correlation. Twelve patients changed correlation sign (75%).

**Conclusion:**

Our results showed that advanced plaque progression had an overall positive correlation with plaque wall stress and a negative correlation with flow shear stress at baseline. However, long-term follow up showed that correlations between plaque progress and mechanical stresses (FSS and PWS) identified for one time period were not re-producible for most cases (>80%). Further investigations are needed to identify the reasons causing the correlation sign changes.

## Introduction

Atherosclerosis development consists of three stages: early initiation, long-term (several decades) slow progression, and some of them final rupture. Low and oscillating blood flow shear stresses (LFSS) have been shown to correlate positively with intimal thickening and atherosclerosis initiation [1-7]. However, the mechanisms governing advanced plaque progression are not well understood. The LFSS hypothesis cannot explain why intermediate and advanced plaques continue to grow under elevated high shear stress conditions [8]. Several groups reported findings contrary to the LFSS hypothesis and suggested the growing importance of searching for other mechanical factors such as plaque wall (structural) stresses (PWS) and new hypotheses for mechanisms governing the plaque progression process [9,10]. In a follow-up study for patients undertaking lipid-lowering therapy (10 patients, 24 months follow-up), Wentzel et al. (2005) reported that flow shear stress did not predict plaque regression [10]. The best predictor of plaque regression was baseline wall thickness. Using in vivo MRI of human carotid data, Tang et al. (2008) reported that 18 out of 21 patients showed negative correlations between human carotid atherosclerotic plaque progression and plaque wall stress on follow-up scan [8]. In the PREDICTION study, Stone et al. also reported that plaque area was a good predictor of change in plaque area (p<0.001), but flow shear stress was not (p=0.32) [11]. In a multi-patient (n=20) intravascular ultrasound (IVUS)-based follow-up study of patients with coronary atherosclerosis, by dividing slices into low (<10 dyn/cm^2^), intermediate (between 10 and 25 dyn/cm^2^), and high (>25 dyn/cm^2^) flow shear stress (FSS) groups and comparing the low and high FSS groups with the intermediate-FSS group, Samady et al. found that low-FSS segments demonstrated greater reduction in vessel (P<0.001) and lumen area (P<0.001), and high-WSS segments demonstrated an increase in vessel (P<0.001) and lumen (P<0.001) area [12]. Using in vivo MRI-based models, Li et al. compared plaque stress conditions from asymptomatic and symptomatic individuals. High stress concentrations were found at the shoulder regions of symptomatic plaques [13].

It should be noted that many authors have used wall shear stress (WSS) and endothelial shear stress (ESS) for flow shear stress. FSS was used in our paper because we are also studying plaque wall stress (PWS) which is the plaque structural maximal principal stress at the lumen wall.

Huge effort has been devoted to identify “mechanisms” governing plaque progression. Correlations between plaque progress and mechanical stresses (including both flow shear stress and plaque wall stress) are considered mechanisms and have been the objectives of many investigations. However, a mechanism has to be reproducible, at least in a statistical sense. That means the observation should remain true for large population and for many observation times. To date, no study was performed to investigate the long term plaque progression correlation behaviors, i.e., if certain correlation behavior (called mechanism) observed in one time interval could be observed in the following time intervals. In this paper, 3D fluid–structure interaction (FSI) models were constructed based on long-term patient follow-up (up to 5–6 years, 2–4 scans, 1–3 time intervals) *in vivo* MRI data. FSI models were used so that we could perform more complete investigations including both plaque wall stress and flow shear stress. Long-term patient follow-up data would enable us to observe if correlations observed at one time interval could be kept in the subsequent time intervals, i.e., if the observed correlation was “reproducible” in a sense. Details are given below.

## Materials and methods

### *In vivo* serial MRI data acquisition and segmentation

**Figure 1:**
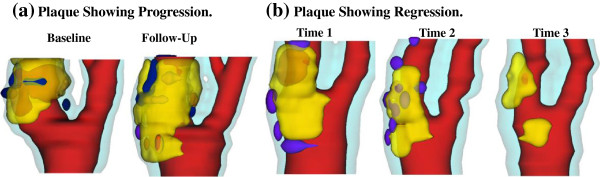
**3D plaque samples re-constructed from *in vivo *MR images showing progression and regression. (a)**: one sample showing plaque growth; **(b)**: one sample showing plaque reduction. Scan time interval: 18 months. Red: lumen; Yellow: lipid; Dark blue: calcification; light blue: outer wall.

After informed consent, serial MRI data of carotid atherosclerotic plaques from 16 patients (15 male, 1 female; age: 59–81, mean=71.9; two patients had L/R carotid MRI usable) were acquired 2–4 times (scan interval: 18 months; 4 scans: 7; 3 scans 8; 2 scans: 1) by the University of Washington (UW) Vascular Imaging Laboratory (VIL) using protocols approved by the UW Institutional Review Board. A total of 38 scan pairs (baseline and follow-up) were formed for progression analysis. MRI scans were conducted on a GE SIGNA 1.5-T whole body scanner using an established protocol (Yuan and Kerwin, 2004). Multi-contrast images in T1, T2, proton density (PD), time-of-flight (TOF), and contrast-enhanced (CE) T1 weighted images of atherosclerotic plaques were generated to characterize plaque tissue composition and luminal and vessel wall morphology [14-16]. Segmentation was performed using a custom-designed computer package CASCADE (Computer-Aided System for Cardiovascular Disease Evaluation) developed by the Vascular Imaging Laboratory at the University of Washington. The slice thickness was 2 mm. Field of view = 160 mm × 160 mm. Matrix size 512 × 512 (the real matrix size was 256 × 256. Images were machine interpolated to 512 × 512). After interpolation, the in-plane resolution was 0.31 × 0.31 mm^2^. Figure 1 gives two examples re-constructed from MRI data showing plaque progression and regression, respectively.

### 3D geometry re-construction and mesh generation

**Figure 2:**
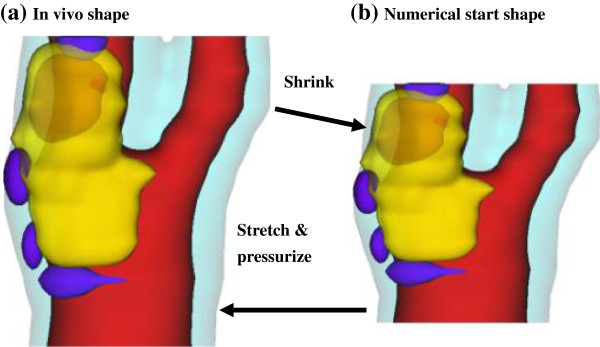
**Illustration of the Shrink-Stretch process.** The total plaque volume should be conserved. Yellow: lipid; Blue: calcification; light blue: outer wall; Red: lumen. **(a)** In vivo shape; **(b)** Numerical starting shape.

Under in vivo conditions, arteries are axially stretched and pressurized. Therefore, in vivo MRI plaque geometry needs to be shrunk axially and circumferentially a priori to obtain the no-load starting geometry (see Figure 2) for computational simulations as our model starts from the no-load geometry with zero stress/strain, zero pressure and no-flow conditions. The shrinkage in axial direction was 9% so that the vessel would regain its *in vivo* length with a 10% axial stretch. Circumferential shrinkage for lumen (about 8-12%) and outer wall (about 2-5%)was determined by trial-and-error so that: 1) total mass of the vessel was conserved; 2) the loaded plaque geometry after 10% axial stretch and pressurization had the best match with the original *in vivo* geometry.

**Figure 3:**
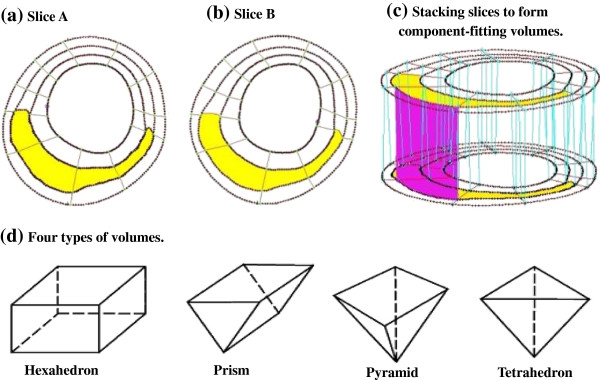
**The component-fitting mesh generation process. (a)-(b)**: two slices with a lipid core inclusion (yellow) and numerically-generated component-fitting curves and surfaces; **(c)** component-fitting volumes formed by connection corresponding areas from stacking adjacent slices; **(d)** Four types of volumes to curve-fit components and complex geometry. Slice distance not to scale.

Because advanced plaques have complex irregular geometries and 3D FSI models involve large deformation and large strain, a geometry-fitting mesh generation technique was developed to generate mesh for these models [17,18]. Using this technique, the 3D plaque and fluid domains were divided into hundreds of small “volumes” to curve-fit the irregular plaque geometries (see Figure 3). Computational meshes for these volumes were then generated automatically by ADINA (ADINA R & D, Inc., Watertown, MA, USA), a commercial finite element software used to solve these FSI models.

### 3D fluid–structure interaction plaque model and solution methods

3D FSI models were constructed using well-established procedures for the 38 pairs (a total of 54 plaque models) to obtain plaque wall stress (PWS) and flow shear stress (FSS) for correlation analysis [17,18]. The artery wall was assumed to be hyperelastic, isotropic, incompressible and homogeneous. The nonlinear modified Mooney-Rivlin model was used to describe the material properties of the vessel wall [19,20]. The strain energy function was given by,

(1) $$W=c_{1}\left(\;I_{1}–3\right)+c_{2}\left(\;I_{2}–3\right)+D_{1}\left[\exp\left(D_{2}\left(\;I_{1}–3\right)\right)–1\right],$$

(2) I1=∑Cii,I2=12I12–CijCij,

where I_1_ and I_2_ are the first and second strain invariants, **C** =[C_ij_] = **X**^T^**X** is the right Cauchy-Green deformation tensor, c_i_ and D_i_ are material parameters chosen to match experimental measurements and the current literature [21,22]. Parameter values used in this paper: vessel/fibrous cap, *c*_1_=36.8 KPa, *D*_1_=14.4 KPa, *D*_2_=2; calcification, c_1_=368 KPa, D_1_=144 KPa, D_2_=2.0; lipid-rich necrotic core, *c*_1_=2 KPa, *D*_1_=2 KPa, *D*_2_=1.5; loose matrix, *c*_1_=18.4 KPa, *D*_1_=7.2 KPa; *D*_2_=1.5. *c*_2_ = 0 was set for all materials [17].

Blood flow was assumed to be laminar, Newtonian, viscous and incompressible. The incompressible Navier–Stokes equations with arbitrary Lagrangian–Eulerian (ALE) formulation were used as the governing equations. A no-slip condition, natural traction equilibrium boundary condition and continuity of displacement were assumed on the interface between solid and fluid. Inlet and outlet were fixed (after initial pre-stretch) in the longitudinal (axial) direction, but allowed to expand/contract with flow otherwise. Patient-specific systolic and diastolic pressure conditions from the last hospital admission were used as the maximum and minimum of the imposed pulsatile pressure waveforms at the inlet and outlet of the artery. Details of the FSI model were given in Tang, et al. (2004, 2009) [17,20].

The 3D FSI models were solved by ADINA, using unstructured finite element methods for both fluid and solid domains. Nonlinear incremental iterative procedures were used to handle fluid–structure interactions. The governing finite element equations for both solid and fluid models were solved by Newton–Raphson iteration method. More details of the computational models and solution methods can be found in Tang et al. (2004, 2009) and Bathe (2002) [18-20]. Plaque wall stress and flow shear stress data corresponding to peak systolic pressure were recorded for analysis.

### Plaque progression measurements and data extraction for correlation analysis

For each scan pair, slices from the baseline (Time 1, or T1) and follow-up (Time 2, or T2; baseline-follow up pairs can also be T2-T3 and T3-T4) scans were matched using the carotid bifurcation as the registration reference. Only matched common carotid artery (CCA) and internal carotid artery (ICA) slices were chosen for analysis. For each matched slice, 100 evenly-spaced points from the lumen were selected and vessel wall thickness, PWS, and FSS from 3D FSI model solutions at each point for baseline and follow-up were obtained for analysis. For the 38 pairs, 400–1000 matched data points for each plaque were obtained for correlation analysis. Plaque progression at each data point was expressed by vessel wall thickness increase (WTI) defined as

(3) WTI=WallThicknessatfollow−up–WallThicknessatbaseline.

### Statistical analysis

The Linear Mixed-Effects (LME) models [23] were used to study the correlation between WTI and the predictors (PWS and FSS) at the initial time point of each time pair. The measured points are nodes, and it seems reasonable to capture the dependence among nodes based on their 3-dimensional spatial locations on the vessel. The models are specified as follows.

For individual patients, the LME model was defined as

(4) $$y_{\mathrm{ij}}=\beta_{0}+\beta_{1}x_{\mathrm{ij}}+\in_{\mathrm{ij}},$$

where *y*_*ij*_ is the WTI value at the *i*th node on the *j*th slice, *x*_*ij*_ is the corresponding value of FSS (or PWS). *β*_0_ and *β*_1_ are the fixed effects of the predictor for the baseline and the changing rate of WTI, respectively. The spatial dependence structure among *y*_*ij*_ is accounted for by the exponential isotropic variogram model in 3D space, which is analogous to the autoregressive model in 1D space [23]. Specifically, the vector of random error terms (*∈*_*ij*_) follows a joint Gaussian distribution with mean 0, and the correlation between $\in_{i_{1}}{}_{j_{1}}$ and $\in_{i_{2}}{}_{j_{2}}$ is assumed an exponential function *ϕ*^s^, where *s* is the Euclidean distance between the 3-dimensional spatial locations of the two nodes on the vessel, *ϕ* is the correlation parameter to be estimated in the model fitting by restricted maximum likelihood (REML) algorithm. The null hypothesis that no correlation exists between WTI and FSS (or PWS) indicates *β*_1_ = 0. A small p-value of the Student’s t-test for the coefficient [23] provides a strong statistical evidence to reject the null and accept the existence of correlation.

To study the correlation between WTI and FSS (or PWS) based on the cohort of patients, the patients were grouped together according to the first (T1-T2), the second (T2-T3), and the third (T3-T4) time-pairs. For all available patients at a particular time pair, the LME model is defined as

(5) $$y_{\mathrm{ijk}}=\beta_{0}+\beta_{1}x_{\mathrm{ijk}}+\;b_{k}+\in_{\mathrm{ijk}},$$

where *y*_*ijk*_ is the WTI value at the *i*th node on the *j*th slice of the *k*th patient, *x*_*ijk*_ is the corresponding value of FSS (or PWS). *β*_0_ and *β*_1_ have the same meanings as those in equation (4). For the *k*th patient, random effect *b*_*k*_ follows a Gaussian distribution with mean 0, which models the clustering dependence of WTI values within this patient. The vector of error terms (*∈*_*ijk*_) follows a joint Gaussian distribution with mean 0. The patients are assumed to be independent so the correlation between the error terms from different patients is 0. The correlation between $\in_{i_{1}}{{}_{j_{1}}}_{k}$ and $\in_{i_{2}}{}_{j_{2}k}$ from the same *k*th patients is assumed an exponential function $\phi_{k}^{s}$, where *s* is the Euclidean distance between the 3D spatial locations, *ϕ*_*k*_ is the patient-specific correlation parameter to be estimated in model fitting. To test the correlation, we again use the p-value for testing *β*_1_ = 0.

To assess the above 3D spatial LME models, we randomly permuted the response WTI values and then calculated the p-values. Such a permutation breaks potential correlations between WTI and FSS (or PWS), so the corresponding p-value is expected to follow a Uniform (0, 1) distribution. Our results verified that the empirical distributions of these p-values after permutations were close to such expectation, which evidenced that our models have the type I error well controlled at the level of individual hypothesis test. That is, these models are appropriate in fitting the correlations between WTI and FSS (or PWS), and the observed small p-values are reliable to indicate the significance of the correlations. We controlled the type I error rate at 0.05.

To measure the direction and magnitude of the linear correlations between WTI and FSS (or PWS), we applied a dependence-adjusted correlation coefficient *r* based on the LME models to account for the spatial dependence structure among the nodes by the exponential isotropic variogram model in 3D space [23], which is described above for models (4) and (5). This *r* is analogous to the Pearson’s correlation coefficient that is valid only for independent observations. Specifically, note that the Pearson’s correlation coefficient between *x* and *y* can be written as:

(6) $$\hat{\mathrm{cor}}\left(x,y\right)=\hat{\beta }\sqrt{\frac{\hat{\mathrm{var}}\left(x\right)}{\hat{\mathrm{var}}\left(y\right)}},$$

where $\hat{\mathrm{var}}\left(x\right)$ and $\hat{\mathrm{var}}\left(y\right)$ are the sample variances, and $\hat{\beta }$ is the estimated slope coefficient by fitting *x* to *y* with a simple regression that requires independence among the observations. Now, since the observations of FSS (or PWS) are dependent, Pearson’s correlation coefficient cannot be directly used for measuring the correlation between FSS (or PWS) and WTI. However, following the same idea as Pearson’s correlation coefficient, we can apply a dependence-adjusted correlation coefficient *r* by

(7) $$r={\hat{\beta }}_{1}\sqrt{\frac{\hat{\mathrm{var}}\left(x\right)}{\hat{\mathrm{var}}\left(y\right)}},$$

where ${\hat{\beta }}_{1}$ is the estimated slope coefficient by fitting FSS (or PWS) to WTI with the LME model (4) or (5). Because ${\hat{\beta }}_{1}$ is estimated in the models that have adjusted the 3D spatial dependence structure among the observations of FSS (or PWS), *r* is the dependence-adjusted correlation coefficient.

## Results

**Figure 4:**
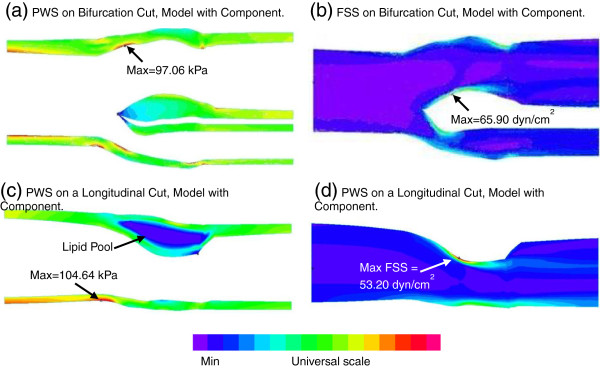
**Plots of PWS and FSS distributions on a bifurcation cut surface and a longitudinal cut surface from FSI model of the plaque sample given in Figure **1**showing PWS and FSS behaviors. (a)** Plaque wall stress on the bifurcation cut; **(b)** flow shear stress on the bifurcation cut; **(c)** plaque wall stress on a longitudinal cut; **(d)** flow shear stress on a longitudinal cut.

Plots of plaque wall stress and flow shear stress from one plaque sample are given in Figure 4 to show the general PWS and FSS behaviors. Correlation results are given below.

### Plaque progression (WTI) correlates positively with plaque wall stress at baseline

Table 1 summarizes correlation results between WTI and PWS at baseline. Out of the 38 scan pairs, 22 pairs showed positive correlation, 1 pair showed negative correlation, and 15 pairs showed no correlation. The overall correlations for the three groups were all positive (Scan 1-Scan 2, 16 pairs; Scan 2-Scan 3, 15 pairs; Scan 3-Scan 4: 7 pairs).

**Table 1:** Correlation summary between WTI and PWS at baseline

***Patient***	***Nodes***	*** TP1-TP2***		***Nodes***	*** TP2-TP3***		***Nodes***	*** TP3-TP4***	
		*** p***	*** r***		*** p***	*** r***		*** p***	*** r***
P1	800	**0.0000**	**0.6048**	800	**0.0019**	**0.0972**			
P2	800	**0.0000**	**0.2946**	900	**0.0000**	**0.0889**	900	0.1063	−0.0508
P3	700	**0.0000**	**0.1222**	800	**0.0014**	**0.0573**	800	**0.0042**	**0.0904**
P4	700	**0.0000**	**0.1512**	700	0.6773	−0.0108			
P5	900	**0.0000**	**0.1889**	900	**0.0066**	**0.0600**			
P6	800	**0.0020**	**0.0449**	800	**0.0000**	**0.1304**	800	0.1876	−0.0249
P7	700	0.1892	0.0160	400	0.3004	0.0266			
P8	400	0.8234	0.0049	900	**0.0038**	**0.0536**			
P9	700	**0.0053**	**0.0422**	700	0.3353	−0.0258			
P10	400	**0.0279**	**0.0244**						
P11	800	**0.0080**	**0.0519**	900	0.1847	−0.0228			
P12	800	**0.0000**	**0.1727**	800	0.0633	0.0323	800	0.0655	0.0477
P13	600	**0.0000**	**0.2123**	600	0.8811	0.0051	500	**0.0000**	**0.3827**
P14	700	0.2534	−0.0199	700	0.1829	0.0393	600	**0.0149**	**−0.0793**
P15	1000	0.3021	−0.0162	1000	**0.0006**	**0.0644**			
P16	900	0.1409	−0.0446	800	**0.0000**	**0.1858**	800	**0.0001**	**0.1129**
All	T1-T2	0.0000	0.0570	T2-T3	0.0000	0.0498	T3-T4	0.0130	0.0283
Total									
P=22		16 pairs	P=11		15 pairs	P= 8		7 pairs	P=3
N=1			N=0			N=0			N=1
NS=15			NS=5			NS=7			NS=3

P=Positive; N=Negative; NS=No Significance.

Bold faced cases are cases of statistical significance.

### Only 16 pairs out of 38 showed negative correlation between WTI and FSS at baseline

Table 2 summarizes correlation results between WTI and FSS at baseline. Out of the 38 scan pairs, 16 pairs showed negative correlation, 1 pair showed positive correlation and 21 pairs showed no correlation. The overall correlations for the three pairing groups were all negative, even though the values of the dependence-adjusted correlation coefficient *r* were small. Correlation between FSS and WTI was weaker than that between PWS and WTI.

**Table 2:** Correlation summary between WTI and FSS at baseline

***Patient***	***Nodes***	***TP1-TP2***		***Nodes***	***TP2-TP3***		***Nodes***	***TP3-TP4***	
		***p***	***r***		***p***	***r***		***p***	***r***
P1	800	**0.0000**	**0.7795**	800	**0.0048**	**−0.1168**			
P2	800	**0.0003**	**−0.1432**	900	0.7463	0.0132	900	0.5646	−0.0157
P3	700	0.0787	−0.0495	800	0.2754	−0.0303	800	**0.0000**	**−0.1859**
P4	700	0.8717	0.0074	700	**0.0316**	**−0.0761**			
P5	900	**0.0026**	**−0.1257**	900	0.0535	−0.0538			
P6	800	0.3243	0.0256	800	0.5825	−0.0165	800	0.0757	−0.0459
P7	700	**0.0015**	**−0.1208**	400	**0.0002**	**−0.1705**			
P8	400	**0.0051**	**−0.1524**	900	0.0909	0.0553			
P9	700	**0.0000**	**−0.2104**	700	**0.0012**	**−0.1732**			
P10	400	**0.0003**	**−0.0749**						
P11	800	0.4927	0.0207	900	**0.0003**	**−0.1034**			
P12	800	**0.0000**	**−0.0900**	800	0.2037	−0.0482	800	0.1904	0.0376
P13	600	0.1071	−0.0864	600	**0.0281**	**−0.0865**	500	0.6058	0.0355
P14	700	0.6729	0.0127	700	0.4306	−0.0322	600	0.0788	−0.0591
P15	1000	0.8519	−0.0044	1000	0.4461	0.0220			
P16	900	0.5383	0.0179	800	**0.0071**	**−0.0906**	800	**0.0082**	**−0.0853**
**All**	**T1-T2**	**0.0350**	**−0.0182**	T2-T3	**0.0003**	**−0.0308**	T3-T4	**0.0001**	**−0.05210**
**Total**									
P=1		16 pairs	P=1		15 pairs	P=0		7 pairs	P=0
N=16			N=7			N=7			N=2
NS=21			NS=8			NS=8			NS=5

P=Positive; N=Negative; NS=No Significance.

Bold faced cases are cases of statistical significance.

### Most patients changed correlation sign during the long-term follow-up study

Table 3 summarized the correlation signs for the 38 pairs grouped into three groups: Period 1: Scan 1 to Scan 2; Period 2: Scan 2 to Scan 3; Period 3: Scan 3 to Scan 4. For correlations between plaque progression and plaque wall stress, 13 patients (81.25%) either had 1 or 2 times sign changes (from +/− to no significance is taken as a change) or showed no significant correlation. Only 3 patients (18.75%) kept their positive correlation sign. For correlations between plaque progression and flow shear stress, 14 patients (87.5%) had 1 or 2 times sign changes. Only 2 patients (12.5%) kept their negative sign.

**Table 3:** Correlation sign change summary between WTI and PWS at baseline

**Pts**	**WTI vs. PWS**			**Sign kept**	**WTI vs. FSS**			**Sign kept**
	**T1-T2**	**T2-T3**	**T3-T4**		**T1-T2**	**T2-T3**	**T3-T4**	
P1	**+**	+		Yes	**+**	**−**		
P2	**+**	+	N		**−**	N	N	
P3	**+**	+	**+**	Yes	N	N	**−**	
P4	**+**	N			N	**−**		
P5	**+**	+		Yes	**−**	N		
P6	**+**	+	N		N	N	N	
P7	N	N			**−**	**−**		yes
P8	N	+			**−**	N		
P9	+	N			**−**	**−**		yes
P10	+				**−**			
P11	**+**	N			N	**−**		
P12	**+**	N	N		**−**	N	N	
P13	**+**	N	**+**		N	**−**	N	
P14	N	N	**−**		N	N	N	
P15	N	+			N	N		
P16	N	+	**+**		N	**−**	**−**	
Patients kept correlation sign				**3**	Patients kept correlation sign			**2**

+: positive correlation; −: negative correlation; N: no correlation or no significance.

## Discussion

### Re-thinking about mechanisms governing advanced plaque progression

A “mechanism” governing a physical or biological process should be something that is true for a majority of events from any given observations, both in terms of samples such as patients and observation time intervals. Then the “mechanism” could be used to predict future trends and possible clinical events, and the prediction should be true at least statistically. There have been huge efforts seeking mechanisms governing plaque progression. It is intuitively natural that low and oscillating flow shear stress would create more favorable flow conditions for cell adhesion and atherosclerosis initiation. Recent advance of medical imaging technology made it possible to track patients non-invasively, obtain patient-specific plaque progression data and quantify possible correlations between plaque progression and various factors such as flow shear stress and plaque wall stress. While the research community has yet to come to a consensus, it is becoming clearer that correlations between advanced plaque progression and mechanical stresses, if they exist, may not be as strong as we thought they were. Results from this study indicated that more than 80% of the patients could not hold their correlation sign over time. If we insist on finding some mechanisms which remained true for all observation intervals for the same patient, those “mechanisms” would be applicable only to 18.75% patients for a positive correlation between plaque progression and plaque wall stress, and 12.5% patients for negative correlation between plaque progression and flow shear stress.

Our results give strong motivations to seeking other mechanisms governing plaque progression, or investigating the reasons causing the correlation sign change. Use of lipid-lowering medications such as statin could be a contributing factor. Diet, cholesterol, mental stress, sudden change of life style, or other disease or illness could all be contributing factors. Growth of plaques depends not only on stress and strain, but a complex biology and physiology environments. Investigation with more possible factors included may lead to new findings.

### Limitations

We are limiting our research to the correlation study between plaque progression and mechanical stresses (FSS and PWS). Other risk factors such as stenosis severity, lipid-rich necrotic cores, and intraplaque hemorrhage will be studies in our subsequent investigations. Other model limitations include: a) the use of an isotropic material model for the vessel because patient-specific anisotropic material properties were not available in vivo [24]; b) flow was assumed laminar because the average stenosis severity (by diameter) of the 54 plaques was 50% and laminar flow assumption was considered acceptable at this level [25]; c) arm systole and diastole pressures taken at scan visit were used to scale the pressure profile used in the simulations since pressure conditions right at the location of the plaque were not available; d) effect of statin use and presence of intraplaque hemorrhage (IPH) were not included in the current study due to lack of complete patient data. Development of IPH would be expected to increase FSS on follow-up scans due to progression in luminal narrowing, but result in continued increase in wall thickness. We are continuing our effort and results will be reported as they become available.

## Competing interests

Other than the grants listed in the acknowledgement section, the authors declare that they have no other competing interest.

## Authors’ contributions

DT and CY (Yang) were responsible for computational modeling and data analysis part. CY (Yuan), GC, and TH were responsible for the MRI and histology data acquisition and the segmentation part. ZW was responsible for the statistical analysis part. All authors 1) have made substantial contributions to conception and design, or acquisition of data, or analysis and interpretation of data; 2) have been involved in drafting the manuscript or revising it critically for important intellectual content; and 3) have given final approval of the version to be published. Each author has participated sufficiently in the work to take public responsibility for appropriate portions of the content.

## Authors’ information

Tang’s group has been publishing image-based modeling work in recent years. For more information, please visit Tang’s website: http://users.wpi.edu/~dtang/.

Dr. Yuan’s group and their lab (Vascular Imaging Laboratory, University of Washington) have been developing MR imaging methods and have published extensively in this area. Website: http://www.rad.washington.edu/research/our-research/groups/vil.
